# Development of the Dietary Practices and Food Safety Literacy Scale for Older Adults

**DOI:** 10.3390/nu17213354

**Published:** 2025-10-24

**Authors:** Ye-Rin Lee, Gi-Moon Nam, Young-Sun Kim, Hye-Ri Shin, Yoo-Kyung Park, Ji-Hye Mun, Su-Hyeun Cho, Hee-Sook Lim

**Affiliations:** 1 Department of Gerontology, AgeTech-Service Convergence Major, Graduate School of East-West Medical Science, Kyung Hee University, Yongin 17104, Republic of Korea; chr021@khu.ac.kr (Y.-R.L.); skarlans52@naver.com (G.-M.N.); ysunkim@khu.ac.kr (Y.-S.K.); zisoa@khu.ac.kr (H.-R.S.); 2 Department of Medical Nutrition, Graduate School of East-West Medical Science, Kyung Hee University, Yongin 17104, Republic of Korea; ypark@khu.ac.kr; 3 Dietary and Nutritional Safety Policy Division, Food and Consumer Safety Bureau, Ministry of Food and Drug Safety, Cheongju 28159, Republic of Korea; jhmun@korea.kr (J.-H.M.); whtngus156@korea.kr (S.-H.C.)

**Keywords:** healthy aging, literacy, older adult, food safety, self-care

## Abstract

Background/Objectives: This study aimed to develop and validate a Dietary Practices and Food Safety Literacy Scale comprehensively assessing competencies among Korean older adults in healthy dietary practices, hygiene, and food safety. Methods: Item development was informed by a literature review, national dietary guidelines, and existing literacy frameworks. Content validity was reviewed by a 10-member expert panel using the Delphi method. Construct validity was tested using exploratory factor analyses (EFA) and confirmatory factor analyses (CFA), and reliability was assessed through Cronbach’s α, composite reliability (CR), and average variance extracted (AVE). Results: EFA identified three factors—management, decision-making, and moderation competencies—comprising 13 items. Internal consistency was acceptable (α = 0.69–0.83), and CFA supported the three-factor structure (CFI = 0.919, RMSEA = 0.087). CR values exceeded 0.70, and AVE were close to or exceeded the recommended threshold. Conclusions: The scale demonstrates sound psychometric properties and provides a practical tool for identifying competency gaps in Dietary Practices and Food Safety Literacy. Its application may guide tailored health education and community-based interventions to promote healthy aging and support public health strategies in aging societies. By translating health information literacy into measurable, behavior-oriented domains, this tool bridges the gap between theoretical constructs and practical assessment. It can be incorporated into routine health monitoring, enabling policymakers and practitioners to design evidence-based interventions that enhance older adults’ dietary self-management and food safety awareness.

## 1. Introduction

South Korea has officially entered a super-aged society, with older adults comprising more than 20% of the population in 9 of 17 provinces [1]. This demographic shift has intensified the need for research aimed at supporting longer, healthier lives. Quality of life (QoL) in later life is shaped by multiple factors, including chronic disease status, functional independence, psychological well-being, age, and socioeconomic status [2,3]. In Korea, however, only 13.9% of older adults are free from chronic disease, while 63.9% live with multimorbidity [4], driving healthcare expenditures to 44% of national spending in 2023—2.5 times the average per capita expenditure [5].

As the burden of chronic conditions grows, older adults are becoming increasingly proactive in managing their health, seeking information beyond traditional healthcare providers [6]. This shift underscores the importance of health literacy—the capacity to obtain, process, and understand health information for informed decision-making [7,8]. A variety of instruments have been developed to assess health literacy, reflecting different dimensions of Nutbeam’s framework—functional, interactive, and critical literacy [9]. Early measures such as the Rapid Estimate of Adult Literacy in Medicine (REALM) [10] and the Test of Functional Health Literacy in Adults (TOFHLA) [11] primarily focused on functional literacy, evaluating reading comprehension and numeracy skills in clinical settings. Although these tools laid the foundation for health literacy assessment, they are largely confined to print-based medical contexts and overlook communication, evaluation, and decision-making competencies required for managing health behaviors in daily life. The Newest Vital Sign (NVS) [12] expanded the concept to nutrition labeling but remains limited to basic comprehension tasks.

Subsequent instruments, including the Communicative and Critical Health Literacy Scale (CCHL) [13], the Health Literacy Questionnaire (HLQ) [14], and the European Health Literacy Survey Questionnaire (HLS-EU-Q) [8], broadened the conceptual scope by integrating interactive and critical dimensions. These frameworks emphasized communication, appraisal, and empowerment, aligning with Nutbeam’s higher-order literacy levels. However, their comprehensive structure often involves lengthy and cognitively demanding items, limiting feasibility among older adults. Similarly, domain-specific tools such as the Food Literacy Assessment Tool (FLAT) [15] and the Nutrition Literacy Assessment Instrument (NLit) [16] address the practical aspects of food choice and nutrition knowledge, yet they typically isolate singular competencies—such as food preparation, selection, or label reading—rather than capturing the integrated processes of managing, deciding, and regulating dietary behaviors.

Building upon these conceptual foundations, the Dietary Practices and Food Safety Literacy Scale developed in this study extends the scope of existing measures by integrating functional, interactive, and critical literacy elements into a coherent, practice-based framework tailored for older adults. Unlike prior tools that primarily assess comprehension or knowledge recall, the Dietary Practices and Food Safety Literacy Scale incorporates behaviorally oriented items that reflect real-life processes of dietary information use—including seeking, evaluating, and applying health and nutrition information. This structure enables a more comprehensive assessment of information application and self-regulation competencies, which were not fully captured by existing nutrition or food literacy instruments such as the NLit and FLAT, thereby operationalizing health literacy within a practical, action-centered context.

Aging also brings complex and multidimensional health needs [17]. Multiple chronic conditions are often accompanied by dietary changes, poor nutritional status [18], reduced physical activity [19], and frailty [20]. Additionally, many older adults demonstrate vulnerabilities in food safety practices—including inadequate knowledge of storage temperatures, expiration dates, and hygiene [21,22]—risks that are further compounded by age-related immune decline.

Given Korea’s rapid demographic transition and these interrelated challenges, there is an urgent need for a comprehensive tool to assess older adults’ competencies in dietary practices, hygiene, and food safety. Therefore, this study aimed to develop and validate such a scale for Korean older adults, designed to identify competency gaps, guide tailored interventions, and inform policies that promote healthy and independent aging in Korea and other super-aged societies.

## 2. Materials and Methods

### 2.1. Study Procedures

**Figure 1:**
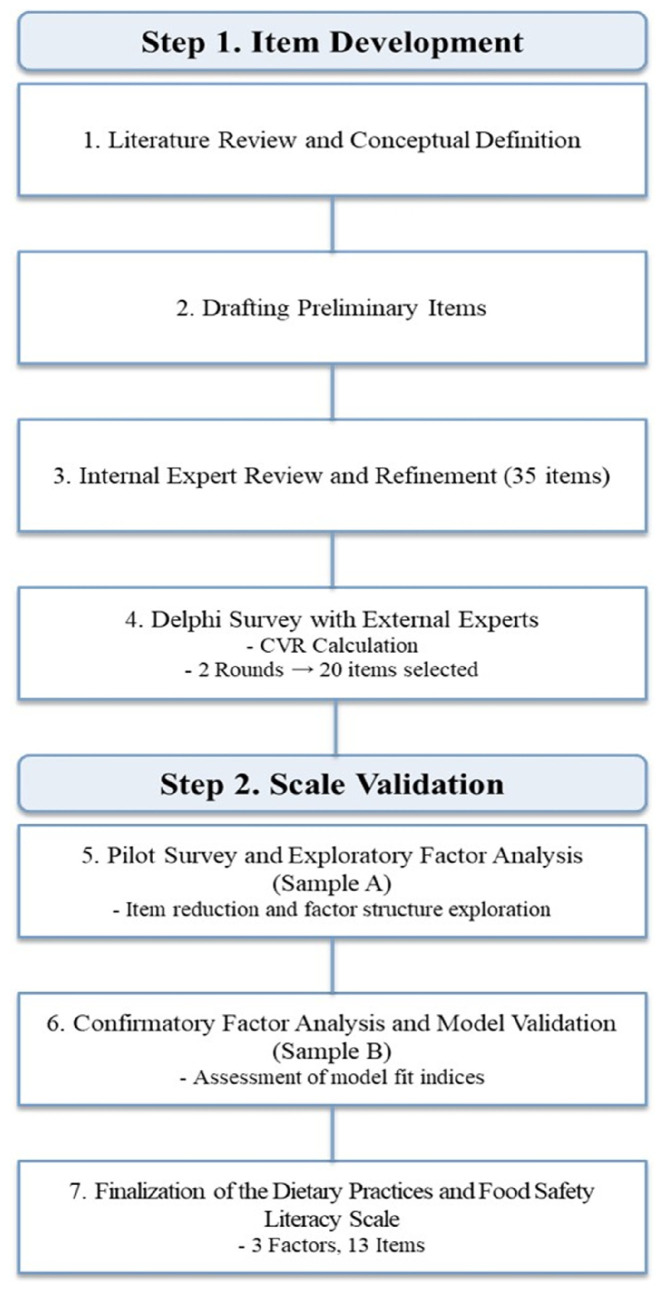
Development process of the Dietary Practices and Food Safety Literacy Scale.

The study procedures were broadly divided into 2 main phases: item development and scale validation (Figure 1). Based on established item development protocols from previous studies, the conceptual framework and preliminary items of the Dietary Practices and Food Safety Literacy Scale for older adults were identified and selected. The items were refined through a content validity assessment conducted by a panel of experts. Subsequently, the revised preliminary items were administered to a sample of community-dwelling older adults to assess construct validity and reliability of the scale. Finally, the Dietary Practices and Food Safety Literacy Scale was statistically validated.

Participants were informed about the study aims and procedures before providing written informed consent. Participation was voluntary, and all responses were kept anonymous and confidential. The study followed the principles of the Declaration of Helsinki and was approved by the Institutional Review Board (IRB) of Kyung Hee University (approval no. KHGIRB-24-369).

### 2.2. Item Development of the Dietary Practices and Food Safety Literacy Scale

As a preliminary step toward developing the Dietary Practices and Food Safety Literacy Scale, a comprehensive literature review was conducted to establish the theoretical definitions of relevant concepts. “Dietary practices” was conceptualized as an inclusive process encompassing the production, selection, preparation, consumption, and disposal of food, as derived from the analysis of lifecycle-specific dietary guidelines provided by organizations such as the Dietary Education Support Center and the Ministry of Food and Drug Safety in Korea, as well as a study by Kim [23]. Another key concept, “hygiene and food safety,” was defined in accordance with the World Health Organization (WHO) as encompassing all measures necessary to ensure the safety and integrity of food throughout the entire process, from cultivation and production to manufacturing and final consumption [24].

In addition, related scales were reviewed to support the conceptual definition of dietary practices and food safety by analyzing the key conceptual elements identified in existing measurement tools. According to previous studies, health literacy is defined as a set of cognitive and social skills comprising the motivation and ability of individuals to access, understand, and use information to promote and maintain health [25]. Food literacy is conceptualized as a comprehensive process involving food-related planning, management, selection, preparation, and consumption activities, along with the associated knowledge, skills, and behaviors necessary to meet food needs and regulate intake [15]. Nutrition literacy is described as the ability to obtain, process, and understand basic health (nutrition) information and services needed to make informed decisions regarding nutrition and overall health [26]. Based on the aforementioned theoretical background, this study defines the Dietary Practices and Food Safety Literacy as “individuals’ ability to acquire, comprehend, and use information and services necessary for health and food safety throughout the process from meal planning to consumption, applying them in daily life to maintain health”.

To develop preliminary items for the Dietary Practices and Food Safety Literacy Scale, domestic dietary guidelines, relevant policy documents, and existing measurement tools were reviewed. Guided by the WHO’s definition of health literacy, a framework was developed to delineate the scope of item development across 4 key processes—information access, understanding, appraisal, and use—organized into 3 core domains: healthy aging, balanced nutrition, and hygiene and safety. Items associated with Dietary Practices and Food Safety Literacy were extracted and reorganized through internal expert meetings. Preliminary items were further refined by modifying ambiguous expressions, eliminating redundant content, adapting the language for relevance to older adults, and removing unsuitable items, resulting in a preliminary pool of 35 items.

To ensure content validity, a panel of 10 external experts from academic, practical, and field-based backgrounds in food and nutrition convened to conduct a Delphi survey. Each item was evaluated using a five-point Likert scale (1 = strongly disagree to 5 = strongly agree), and qualitative feedback was obtained. Based on the panelists’ evaluations, the content validity ratio (CVR) for each item was calculated according to Lawshe’s method [27]. Following 2 rounds of content validity assessment, 20 items with a CVR greater than 0.62 were finalized to comprise the Dietary Practices and Food Safety Literacy Scale.

Through this process, 20 preliminary items were finalized and subsequently used in the pilot survey and scale validation phase. In Korea, welfare centers located in areas with a high proportion of older adults were contacted, and those that agreed to participate were included in the study. Within these centers, an official recruitment poster containing information about the study was displayed, older adults who received information about the study and voluntarily provided informed consent were recruited through convenience sampling. And then, Data were collected from November to December 2024 to evaluate the validity and reliability of the preliminary items among community-dwelling adults aged 65 years and older. The survey was administered using a self-report questionnaire on a five-point Likert scale (1 = strongly disagree to 5 = strongly agree). Higher total scores indicate higher levels of the Dietary Practices and Food Safety Literacy. Among the collected responses, 224 questionnaires were included in the analysis, excluding 2 with missing data. To minimize potential errors arising from analyzing a single sample, the entire dataset was randomly divided into two groups based on an appropriate sample size criterion. Although the 5–10 participants-per-item rule is commonly used as a guideline for factor analysis [28], methodological research has consistently emphasized that sample adequacy should be evaluated based on data and model characteristics rather than fixed ratio rules. MacCallum et al. [29] emphasized that sample size requirements depend on communalities and the number of variables, and subsequent studies also emphasized that model complexity, factor determinacy, and loading patterns substantially affect the required sample size [30]. In this study, communalities and factor loadings were within an acceptable range, indicating generally adequate conditions for factor analysis.

Exploratory factor analysis (EFA) was conducted with Sample A (*n* = 108) to assess item quality and determine the number of factors, whereas confirmatory factor analysis (CFA) was performed with Sample B (*n* = 116) to validate the structural adequacy of the designed scale. All statistical analyses were performed using IBM SPSS Statistics version 28.0 (IBM Co., Armonk, NY, USA) and AMOS version 29.0 (IBM Co., Armonk, NY, USA).

### 2.3. Validation and Reliability of the Dietary Practices and Food Safety Literacy Scale

First, preliminary descriptive statistics were performed using Sample A to examine the mean, standard deviation, skewness, and kurtosis of each item. Items with a mean score lower than 1.0, higher than 4.0, or with a standard deviation greater than 1.5 were considered for removal because of their low discriminative power [31,32,33]. To assess normality, items were deemed to satisfy the assumption of a normal distribution if the absolute value of skewness was less than 2 and the absolute value of kurtosis was less than 7 [34]. Additionally, inter-item correlations in subscales and item-total correlations were reviewed, with acceptable values set between 0.20 and 0.50 [32]. Items outside this range were considered for removal. Internal consistency was evaluated using Cronbach’s alpha, with values above 0.60 considered acceptable and those above 0.70 regarded as good reliability [35].

EFA was conducted to identify the underlying common dimensions based on the interrelationships among multiple variables. Prior to factor extraction, the suitability of the data for factor analysis was evaluated using the Kaiser–Meyer–Olkin (KMO) measure of sampling adequacy and Bartlett’s test of sphericity. A KMO value close to 1.0 indicates sampling adequacy. Values above 0.90 were considered marvelous, above 0.80 meritorious, above 0.70 middling, above 0.60 mediocre, above 0.50 miserable, and below 0.50 unacceptable [36]. The significance of Bartlett’s test was examined to determine whether the null hypothesis of no correlation among the variables could be rejected. Principal axis factoring was employed for factor extraction. Factor rotation was performed to achieve a simpler and more interpretable factor structure. Given the assumption that these factors are correlated, an oblique rotation method, specifically direct oblimin rotation, was used [37,38]. Items with communalities below 0.30 were considered for removal. Based on the theoretical framework suggesting 3 subdimensions, a maximum of 4 factors were considered. Factor retention decisions were made with reference to Kaiser’s criterion (eigenvalues greater than 1) and inspection of the scree plot, with the cumulative variance threshold set at a minimum of 60%.

Based on the EFA, a CFA was conducted using Sample B to validate the scale’s structure. Structural equation modeling was used to test the structural validity of the Dietary Practices and Food Safety Literacy Scale. Model fit was evaluated using various indices, including the Tucker–Lewis index (TLI), comparative fit index (CFI), goodness of fit index (GFI), root mean square error of approximation (RMSEA), and standardized root mean square residual (SRMR). A TLI and CFI value of 0.90 or higher was considered indicative of good model fit. Regarding RMSEA, smaller values indicated better fit: values less than 0.05 were considered excellent, less than 0.08 acceptable, less than 0.10 marginal, and greater than 0.10 poor [34]. In addition to model fit, convergent and discriminant validities were assessed. Convergent validity was confirmed if standardized factor loadings were greater than 0.50, composite reliability (CR) exceeded 0.70, and the average variance extracted (AVE) was greater than 0.50 [39]. Therefore, the statistical validity and reliability of the Dietary Practices and Food Safety Literacy Scale were verified.

## 3. Results

### 3.1. Exploration of the Factor Structure for the Development of the Dietary Practices and Food Safety Literacy Scale

This was a pilot study, and the main study was conducted to statistically validate the development of the Dietary Practices and Food Safety Literacy Scale for older adults. Table 1 shows the characteristics of Samples A and B used in each phase.

A pilot study was conducted to explore the scale’s item structure and validity of its factor structure. Before analysis, normality tests indicated that the data met the assumptions of normality. Descriptive statistics and internal consistency measures were calculated for each item. The mean scores ranged from 3.46 to 4.56, and the standard deviations ranged from 0.568 to 1.249, indicating acceptable levels of distribution. Items with a mean score exceeding 4.0 included statements such as “I believe that nutritional management is essential for healthy aging,” “I regularly assess my dietary habits and strive to improve unhealthy eating patterns,” and “I always pay attention to hygiene and handle food safely when preparing meals.” Although potential response bias was considered, these items were retained because they contained essential conceptual elements that reflected participants’ attitudes and intentions.

The initial Cronbach’s alpha coefficients for each category were 0.677 for healthy aging, 0.739 for balanced nutrition, 0.814 for hygiene and food safety, and 0.898 for the total scale. These results demonstrated that each of the 3 categories achieved an acceptable level of internal consistency, with α values above 0.60. However, in examining item-total correlations in subscales, 2 items—“I drink a sufficient amount of water daily (at least six glasses)” and “I am aware of organizations where I can seek help when facing meal-related difficulties”—showed correlations below 0.30, indicating low association with their respective subscales. Accordingly, these 2 items were excluded from further analyses.

Following the preliminary item analysis, EFA was conducted using the remaining 18 items that passed the quality assessment. The KMO measure of sampling adequacy was 0.854, and Bartlett’s test of sphericity was significant (*p* < 0.001), confirming the appropriateness of data for factor analysis. Principal axis factoring with direct oblimin rotation is employed, and Table 2 shows the results. Factors were extracted based on Kaiser’s criterion (eigenvalues greater than 1) and inspection of the scree plot with a cumulative variance explanation threshold of at least 60%, resulting in the retention of 3 factors. In the factor structure matrix, when an item was loaded above 0.40 on more than 1 factor, discriminant and convergent validity were assessed by verifying that the difference between the highest and second-highest factor loadings exceeded 0.10.

The items grouped in each factor were as follows: The first factor comprised 7 items: efforts to monitor and improve dietary practices, checking the use-by date, practicing proper handwashing, ability to prepare simple meals independently, preference for eating with others, selecting food safety information from the media, and recognizing the importance of nutrition management in later life. The second factor included 4 items: checking labels when purchasing food or supplements, selecting ingredients beneficial for health maintenance, practicing balanced eating for healthy aging, and seeking health information. Finally, the third factor comprised 2 items: adherence to appropriate meal portions and physical activity levels and practicing regular meal habits.

A total of 3 factors and 13 items were finalized. Although the extracted factor structure differed from the initially proposed conceptual categories, it reflected the multidimensional competencies that constitute the Dietary Practices and Food Safety Literacy among older adults. Accordingly, these factors were named according to their conceptual characteristics. The first factor was designated as “management competencies,” comprising basic abilities associated with the overall dietary practices and personal hygiene management. The second factor was named “decision-making competencies,” representing the cognitive and voluntary abilities to seek, evaluate, and apply health and nutrition information to make appropriate and informed decisions that support healthy living in daily life. representing the voluntary abilities to independently seek information, apply it, and actively use it in daily life. The third factor was termed “moderation competencies,” which included self-regulatory skills for managing meal portions and physical activity levels.

Cronbach’s alpha for each factor was as follows: 0.829 for management competencies, 0.781 for decision-making competencies, and 0.685 for moderation competencies, indicating acceptable internal consistency. The correlation analysis of the 3 factors revealed coefficients ranging from r = 0.253 to r = 0.435, suggesting that the factors maintained an independent structure without excessive intercorrelation.

### 3.2. Validation of the Factor Structure of the Dietary Practices and Food Safety Literacy Scale

The main study was conducted to confirm the factor structure identified in the EFA. CFA was performed to assess the extent to which the relationships between the subfactors and their corresponding observed variables, as specified through EFA, accurately reflected the response patterns of the study participants. Considering potential issues related to item loss and factor interpretability, the three-factor structure was identified as the most stable and theoretically coherent solution. Although two- and four-factor models were also examined, they showed poorer model fit and required the elimination of several key items, which undermined conceptual validity and structural consistency. In contrast, the three-factor model demonstrated satisfactory statistical fit and theoretical coherence and was therefore adopted as the final model, comprising management, decision-making, and moderation competencies. A detailed examination of the model was performed. In addition, modification indices (MI) were examined to improve model fit. High MI values were identified between certain error terms within the same subfactor—particularly between items e1 and e4, e8 and e15, and e9 and e13. Based on theoretical rationale and content similarity, covariance paths were added between these error terms. Following these modifications, the model fit improved [40].

The results of the CFA are presented in Table 3. The major model fit indices were as follows: GFI = 0.874, CFI = 0.919, RMSEA = 0.087, and SRMR = 0.066. Although the RMSEA value indicated a marginal fit, the other indices were within acceptable ranges, suggesting that the overall model exhibited an adequate fit to the response data.

**Figure 2:**
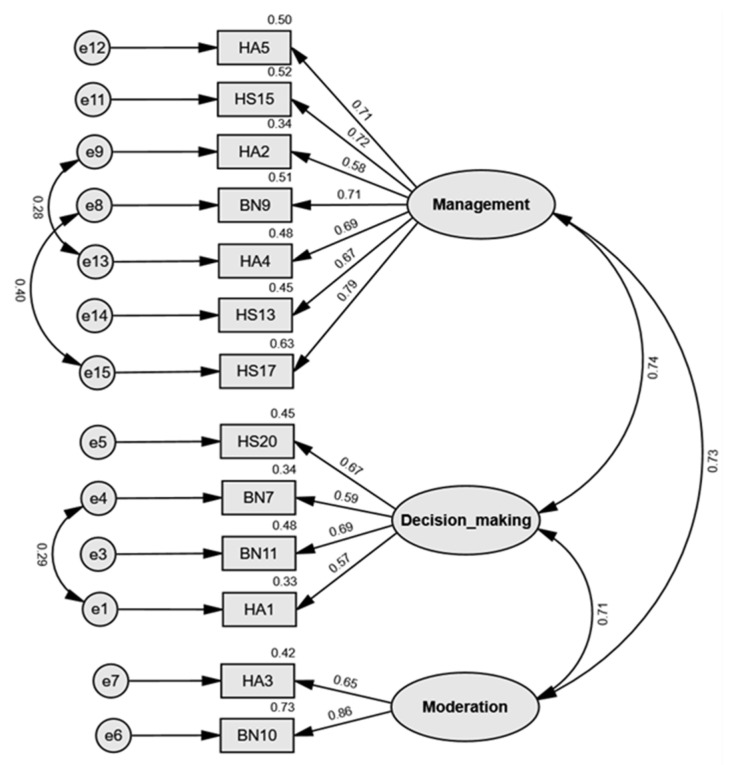
Final 13-item scale structure of the Dietary Practices and Food Safety Literacy Scale. The figure presents three factors: Factor 1 (Management competencies), Factor 2 (Decision-making competencies), and Factor 3 (Moderation competencies).

Figure 2 shows the standardized parameter estimates of the model. All factor loadings were statistically significant at *p* < 0.001, with coefficients ranging from 0.572 to 0.857, confirming that each item reliably measured its corresponding subfactors.

To assess convergent validity, composite reliability (CR) and average variance extracted (AVE) were calculated for each construct based on factor loadings and error variances. The CR values were 0.879 for management competencies (Factor 1), 0.742 for decision-making competencies (Factor 2), and 0.720 for moderation competencies (Factor 3), all exceeding the recommended threshold of 0.60. The AVE values were 0.512, 0.420, and 0.567 for Factors 1, 2, and 3, respectively. Although the AVE for decision-making competencies (0.420) was slightly below the recommended threshold of 0.50, its CR exceeded 0.60, indicating overall acceptable convergent validity according to previous studies [41,42]. As noted by Fornell and Larcker [41], an AVE value below 0.50 suggests that measurement error accounts for more variance than the latent construct itself, implying limited convergence among the indicators. Accordingly, while the decision-making competency factor demonstrated adequate internal consistency, the relatively low AVE reflects a potential limitation that should be taken into account when interpreting the construct validity of this dimension.

Discriminant validity was examined by calculating the confidence intervals for the correlations between the factors, using the formula correlation coefficient ± 2 × standard error (SE). The correlation between Factors 1 and 2 was 0.738 with a confidence interval of 0.566–0.910; between Factors 2 and 3 was 0.712 with an interval of 0.536–0.888; and between Factors 3 and 1 was 0.726 with an interval of 0.500–0.952. None of these intervals included 1.0, thereby confirming that discriminant validity was established among the three factors. Consequently , the Dietary Practices and Food Safety Literacy Scale developed in this study was verified to meet validity and reliability criteria. The complete 13-item scale is provided in Supplement S1.

## 4. Discussion

This study developed and validated the Dietary Practices and Food Safety Literacy Scale for older adults, encompassing three competency domains: management, decision-making, and moderation. The scale assesses practical, action-oriented skills essential for maintaining balanced dietary practices, ensuring food safety, and promoting healthy lifestyle behaviors.

Although the three domains of the Dietary Practices and Food Safety Literacy Scale—management, decision-making, and moderation competencies—were developed empirically from behavioral indicators rather than directly from Nutbeam’s theoretical model [9], they conceptually parallel the functional, interactive, and critical dimensions of health literacy. Management competencies correspond to functional literacy, representing basic understanding and implementation of dietary and hygiene behaviors. Decision-making competencies align with interactive literacy, reflecting active information-seeking, evaluation, and informed decision-making. Moderation competencies relate to critical literacy, encompassing self-regulatory and empowerment capacities to maintain dietary balance and health behaviors.

While these domains do not perfectly mirror Nutbeam’s framework, they collectively represent a comparable developmental continuum of dietary literacy—from understanding, to interaction, to critical control—thus extending the conceptual reach of health literacy into the domains of food safety and daily dietary management. This interpretation is also consistent with Sørensen et al.’s [8] integrated model of health literacy, which views health literacy as a multidimensional process involving the ability to access, understand, appraise, and apply information across diverse health contexts.

Building on this theoretical alignment, each domain of the scale can be further delineated in terms of its practical focus and behavioral scope. The management domain focuses on basic abilities associated with overall dietary practices and personal hygiene management. It reflects individuals’ capacity to plan, organize, and maintain daily eating and hygiene behaviors, emphasizing the behavioral and maintenance components of dietary literacy. The decision-making domain represents the cognitive and voluntary abilities to seek, evaluate, and apply health and nutrition information to make appropriate and informed decisions that support healthy living in daily life. This domain captures the cognitive and motivational aspects of using reliable information to make sound dietary choices. The moderation domain addresses self-regulatory skills for balancing meal portions and physical activity levels, highlighting individuals’ ability to monitor, adjust, and sustain dietary and lifestyle behaviors in alignment with health goals. Together, these domains define the structural framework of Dietary Practices and Food Safety Literacy, integrating informational, behavioral, and self-regulatory capacities that are conceptually aligned with the WHO’s holistic view of health literacy and empowerment [43,44].

From a global perspective, Dietary Practices and Food Safety Literacy Scale was developed and validated among Korean older adults, the scale reflects universal dimensions of dietary literacy that are potentially generalizable across cultures. Nevertheless, certain aspects—such as collective eating norms, culturally shaped food safety awareness, and family-centered dietary practices—may influence how the three competencies manifest in the Korean context. Future research should therefore conduct cross-cultural validation of the scale to examine the cultural relevance and structural equivalence of these domains in Western and other Asian populations. Such efforts would contribute to refining the theoretical understanding of how management, decision-making, and moderation competencies operate across diverse sociocultural settings. Accordingly, while the present validation supports the scale’s conceptual coherence and psychometric soundness within the Korean context, further cross-population validation is warranted before broader generalization.

Most existing health literacy tools for older adults have been adaptations of general instruments or developed for specific disease contexts, such as the Chinese Health Literacy Scale for Chronic Care [45], the Korean version of the European Health Literacy Survey Questionnaire (K-HLS-EU-Q47) [46], and the expanded food literacy tool by So et al. [47]. While these instruments offer valuable insights, they often lack comprehensive coverage of self-management competencies in daily dietary and hygiene practices.

The scale developed in this study advances the field by operationalizing the concept of “health information” into concrete, measurable domains directly relevant to chronic disease prevention and dietary safety management, including balanced dietary intake, healthy eating habits, regular meal patterns, engagement in physical activities, as well as risk perception and preventive behavioral intentions associated with food safety incidents [48,49,50,51,52,53].

Furthermore, these competencies are closely linked to health empowerment, which fosters autonomous decision-making and active engagement in health-promoting behaviors. As reported in Lee and Lim [54], higher health empowerment significantly predicted increased exercise and nutrition management in older adults (β = 0.40, *p* < 0.001), and similar associations have been observed in another research [55]. Recent studies have demonstrated that digital and community-based screening tools are effective in identifying health literacy gaps and promoting self-management among older adults [56,57,58]. Building on this evidence, the Dietary Practices and Food Safety Literacy Scale could be integrated into e-health platforms or community-based health programs to monitor competencies in real time, support personalized feedback, and guide early interventions. By assessing Dietary Practices and Food Safety Literacy, healthcare providers can identify vulnerable competencies and design targeted interventions, ultimately promoting self-management, improving health behaviors, and enhancing quality of life in aging populations.

Although these findings offer meaningful implications for practice and policy, several limitations should be considered when interpreting the results. These include the use of a convenience sample from selected regions and suboptimal fit indices for some model parameters. The overall model fit was acceptable but not optimal (CFI = 0.919, GFI = 0.874, TLI = 0.893, RMSEA = 0.087, SRMR = 0.066). Although the CFI and SRMR met conventional thresholds for acceptable fit, the GFI and TLI were slightly below recommended values, and the RMSEA approached the upper limit of acceptability, indicating that the model fit was adequate but not fully satisfactory. Accordingly, the findings can be viewed as generally supportive of the hypothesized structure, though some caution is warranted in interpretation.

Because participants were recruited through convenience sampling from a limited number of regions, the findings may not fully represent the broader population of older adults in Korea. To address this limitation, Future studies should therefore include participants from more diverse geographic areas to enhance external validity and minimize potential sampling bias. In addition, Future studies should validate the scale across diverse populations, stratifying by gender, socioeconomic status, and health condition, and conduct longitudinal analyses to examine the relationship between Dietary Practices and Food Safety Literacy, and behavioral change.

## 5. Conclusions

The Dietary Practices and Food Safety Literacy Scale developed in this study provides an empirically supported and comprehensive framework for evaluating older adults’ competencies in dietary management, information use, and behavioral regulation. While the current validation demonstrates conceptual coherence and acceptable psychometric performance within the Korean context, these findings should be interpreted as preliminary. Further cross-cultural validation is warranted before broader application to other populations. By translating the abstract concept of “health information” into concrete, measurable domains—balanced dietary intake, healthy eating habits, regular meal patterns, physical activity engagement, and risk perception regarding food safety incidents—the scale captures the practical self-management skills essential for preventing chronic conditions and supporting healthy aging.

In practice, the scale may be utilized as a brief screening or educational assessment tool in community centers, welfare facilities, and primary care clinics to help identify areas of limited competency and guide the design of tailored interventions. Such applications could potentially strengthen older adults’ self-management abilities, enhance problem-solving and dietary decision-making skills, and support sustainable health behavior change, ultimately contributing to reduced healthcare costs and improved quality of life [59,60,61].

At the policy level, the present findings may serve as preliminary evidence to inform the development of national health indicators and community-based programs that foster autonomous health management in rapidly aging societies. Future research should validate the scale across diverse demographic and cultural groups, assess its predictive value for long-term health outcomes, and explore integration with digital health applications to enhance accessibility. Continued refinement and application of this tool are expected to contribute to creating supportive environments that empower older adults to make informed, safe, and balanced dietary decisions.

## Figures and Tables

**Table 1:** Demographic Characteristics of the Participants (*N* = 224).

Variables	Sample A (*n* = 108)	Sample B (*n* = 116)
Area of Residence		
Urban	19 (17.6)	32 (27.6)
Rural	89 (82.4)	84 (72.4)
Gender		
Men	31 (28.7)	44 (37.9)
Women	77 (71.3)	72 (62.1)
Age (yrs) (A)	76.13 ± 5.32	77.67 ± 4.95
Subjective Age (yrs) (B)	73.38 ± 8.89	73.60 ± 7.20
(A)–(B)	2.75 ± 10.36	4.07 ± 8.73
Age Range		
65–74 years	42 (38.9)	30 (25.9)
75–79 years	37 (34.3)	44 (37.9)
80+ years	29 (26.9)	42 (36.2)
Marital Status		
Married	43 (39.8)	45 (38.8)
Separated	0	3 (2.6)
Divorced	21 (19.4)	28 (24.1)
Widowed	42 (38.9)	37 (31.9)
Single	2 (1.9)	3 (2.6)
Household Composition		
Living alone	64 (59.3)	67 (57.8)
With spouse	36 (33.3)	34 (29.3)
With adult children	5 (4.6)	6 (5.2)
With spouse and adult children	2 (1.9)	7 (6.0)
Other family members	1 (0.9)	2 (1.7)
Educational Attainment		
No formal education	5 (4.6)	8 (6.9)
Elementary school graduate	45 (41.7)	53 (45.7)
Middle school graduate	28 (25.9)	24 (20.7)
High school graduate	25 (23.1)	22 (19.0)
College graduate and over	5 (4.6)	9 (7.8)
Monthly Household Income (KRW 10,000)		
<100	68 (63.0)	75 (64.7)
100–199	18 (16.7)	22 (19.0)
200–299	13 (12.0)	10 (8.6)
300–499	6 (5.6)	5 (4.3)
≥500	3 (2.8)	4 (3.4)
Monthly Average Food Expenditure (KRW 10,000)		
≤10	28 (25.9)	31 (26.7)
11–30	28 (25.9)	27 (23.3)
31–49	23 (21.3)	28 (24.1)
≥50	29 (26.9)	30 (25.9)

Note. Data are presented as numbers (%), mean ± standard deviation. KRW = Korean won. Sample A: sample used for exploratory factor analysis (EFA); Sample B: sample used for confirmatory factor analysis (CFA).

**Table 2:** Exploratory Factor Analysis of the Dietary Practices and Food Safety Literacy Scale (Sample A, *n* = 108).

No.	Items	Communalities	Factor Loadings		
			1	2	3
Factor 1. Management Competencies					
HA5	I regularly assess my dietary habits and strive to improve unhealthy eating patterns.	0.618	**0.783**	0.305	0.273
HS15	When I purchase or consume food, I check the use-by date.	0.591	**0.729**	0.506	0.437
HS17	I always wash my hands with the right way to wash my hands before cooking or eating.	0.464	**0.648**	0.463	0.346
BN9	I can prepare food by myself by referring to simple recipes.	0.389	**0.622**	0.261	0.304
HA4	I like to share food with my family, acquaintances, and neighbors, or eat together.	0.427	**0.619**	0.228	0.445
HS13	I can select the right information among the food safety information in the mass media (e.g., TV, Internet, YouTube, books).	0.428	**0.602**	0.455	0.146
HA2	I believe that nutritional management is essential for healthy aging.	0.331	**0.571**	0.200	0.281
Factor 2. Decision-Making competencies					
HS20	I check the food labels (e.g., certification marks, ingredients) when purchasing general foods, dietary supplements, or functional foods.	0.647	0.315	**0.797**	0.098
BN7	I can identify and select food products that support a healthy diet (e.g., low-sugar, low-sodium, high-protein foods).	0.527	0.404	**0.719**	0.216
BN11	I usually have a nutritionally balanced diet with nutrient-rich foods (e.g., fish, meat, eggs, legumes, vegetables, and enough fluids) that help maintain my health as I age.	0.567	0.314	**0.700**	0.430
HA1	I can find helpful and reliable health information (e.g., disease prevention, nutrition, physical activity, healthy aging, and community meal services).	0.315	0.337	**0.550**	0.203
Factor 3. Moderation competencies					
HA3	I tend to keep an appropriate amount of food and physical activity.	0.598	0.409	0.394	**0.743**
BN10	I eat every meal regularly.	0.537	0.453	0.185	**0.710**
Eigen value		-	5.087	1.627	1.150
Explanatory Power (%)		-	39.128	12.517	8.843
Cumulative Variance (%)		-	39.128	51.645	60.488
Cronbach’s alpha		Total 0.864	0.829	0.781	0.685

Note. Factor loadings of the Dietary Practices and Food Safety Literacy Scale items for each factor are shown in boldface. Factor extraction was conducted using principal axis factoring with oblimin rotation. HA = Healthy Aging; BN = Balanced Nutrition; HS = Hygiene and Safety.

**Table 3:** Model Fit Indices for the Dietary Practices and Food Safety Literacy Scale (Sample B, *n* = 116).

χ^2^	*df*	CFI	GFI	TLI	RMSEA	SRMR
110.777 ***	59	0.919	0.874	0.893	0.087	0.066

χ^2^, chi-square test; *df*, degrees of freedom; CFI, comparative fit index; GFI, goodness of fit index; TLI, Tucker–Lewis index; RMSEA, root mean square error of approximation; SRMR, standardized root mean square residual. *** *p* < 0.001.

## Data Availability

The data presented in this study are available upon request from the corresponding author due to privacy restrictions.

## Supplementary Materials

The following supporting information can be downloaded at: https://www.mdpi.com/article/10.3390/nu17213354/s1, Supplement S1: Dietary Practices and Food Safety Literacy Scale.
