# Supplementary material for: Development of the Dietary Practices and Food Safety Literacy Scale for Older Adults
Source: Nutrients. 2025 Oct 24;17(21):3354. doi: 10.3390/nu17213354 (PMC12610686; doi:10.3390/nu17213354)
Supplement: Supplementary file 1 [file nutrients-17-03354-s001.zip › nutrients-3927857-supplementary.pdf]

Supplement S1: Dietary Practices and Food Safety Literacy Scale

The ***Dietary Practices and Food Safety Literacy Scale*** assesses individuals' competencies in dietary and food safety practices that support healthy aging. Please consider your current lifestyle and dietary habits, and select the response that best reflects how each statement applies to you. Indicate how much you agree with each statement or how often it applies to you.

(1 = Strongly disagree / Never ~ 5 = Strongly agree / Always)

No.	Items	Responses Scale				
**1**	I believe that nutritional management is essential for healthy aging.	(5)	(4)	(3)	(2)	(1)
**2**	I tend to keep an appropriate amount of food and physical activity.	(5)	(4)	(3)	(2)	(1)
**3**	I regularly assess my dietary habits and strive to improve unhealthy eating patterns.	(5)	(4)	(3)	(2)	(1)
**4**	I like to share food with my family, acquaintances, and neighbors, or eat together.	(5)	(4)	(3)	(2)	(1)
**5**	I eat every meal regularly.	(5)	(4)	(3)	(2)	(1)
**6**	I can identify and select food products that support a healthy diet (e.g., low-sugar, low-sodium, high-protein foods).	(5)	(4)	(3)	(2)	(1)
**7**	I always wash my hands with the right way to wash my hands before cooking or eating.	(5)	(4)	(3)	(2)	(1)
**8**	When I purchase or consume food, I check the use-by date.	(5)	(4)	(3)	(2)	(1)
**9**	I can prepare food by myself by referring to simple recipes.	(5)	(4)	(3)	(2)	(1)
**10**	I usually have a nutritionally balanced diet with nutrient-rich foods (e.g., fish, meat, eggs, legumes, vegetables, and enough fluids) that help maintain my health as I age.	(5)	(4)	(3)	(2)	(1)
**11**	I can find helpful and reliable health information (e.g., disease prevention, nutrition, physical activity, healthy aging, and community meal services).	(5)	(4)	(3)	(2)	(1)
**12**	I can select the right information among the food safety information in the mass media (e.g., TV, Internet, YouTube, books).	(5)	(4)	(3)	(2)	(1)
**13**	I check the food labels (e.g., certification marks, ingredients) when purchasing general foods, dietary supplements, or functional foods.	(5)	(4)	(3)	(2)	(1)

Scoring

1. Count the number of responses for each response option.
2. Multiply each count by the corresponding score.
3. Calculate the total score.

**Number of Responses**					
**Score**	**× 5**	**× 4**	**× 3**	**× 2**	**× 1**
**Total Points (per Category)**					
**Overall Total Score**	______________ points				

Note. This scale is protected by copyright. Permission to use, adapt, or reproduce the scale must be obtained from the developing author (correspondence).
